# A Novel Model of Cancer Drug Resistance: Oncosomal Release of Cytotoxic and Antibody-Based Drugs

**DOI:** 10.3390/biology9030047

**Published:** 2020-03-05

**Authors:** Takanori Eguchi, Eman Ahmed Taha, Stuart K. Calderwood, Kisho Ono

**Affiliations:** 1 Department of Dental Pharmacology, Graduate School of Medicine, Dentistry and Pharmaceutical Sciences, Okayama University, Okayama 700-8525, Japan; pj7l8pfb@s.okayama-u.ac.jp; 2 Advanced Research Center for Oral and Craniofacial Sciences, Graduate School of Medicine, Dentistry and Pharmaceutical Sciences, Okayama University, Okayama 700-8525, Japan; 3 Department of Medical Bioengineering, Graduate School of Natural Science and Technology, Okayama University, Okayama 700-8530, Japan; 4 Department of Biochemistry, Ain Shams University Faculty of Science, Cairo 11566, Egypt; 5 Department of Radiation Oncology, Beth Israel Deaconess Medical Center, Harvard Medical School, Boston, MA 02215, USA; scalderw@bidmc.harvard.edu; 6 Department of Oral and Maxillofacial Surgery, Okayama University Hospital, Okayama 700-0914, Japan; de20012@s.okayama-u.ac.jp

**Keywords:** extracellular vesicle (EV), exosome, oncosome, drug resistance, epithelial-mesenchymal transition (EMT), heat shock protein (HSP), cell stress response, resistance-associated secretory phenotype (RASP), hypoxia, acidosis, tumor immunology

## Abstract

Extracellular vesicles (EVs), such as exosomes or oncosomes, often carry oncogenic molecules derived from tumor cells. In addition, accumulating evidence indicates that tumor cells can eject anti-cancer drugs such as chemotherapeutics and targeted drugs within EVs, a novel mechanism of drug resistance. The EV-releasing drug resistance phenotype is often coupled with cellular dedifferentiation and transformation in cells undergoing epithelial-mesenchymal transition (EMT), and the adoption of a cancer stem cell phenotype. The release of EVs is also involved in immunosuppression. Herein, we address different aspects by which EVs modulate the tumor microenvironment to become resistant to anticancer and antibody-based drugs, as well as the concept of the resistance-associated secretory phenotype (RASP).

## 1. Introduction

Recent studies have unveiled the existence of and significant biological roles for extracellular vesicles (EVs). EVs are nano-particles surrounded by lipid membranes, containing a variety of molecular cargos such as proteins, small and large RNAs, DNA, lipids, glycans, minerals, and metabolites that are thus secreted by cells [1,2,3,4,5]. Earlier studies have classified the range of EVs into exosomes (50–200 nm), ectosomes (100–1000 nm; also known as microvesicles) [6,7,8], and apoptotic bodies (1–10 μm) based on their mechanisms of generation and release, while additional types of EVs have been reported, consisting of oncosomes (oncogenic EVs) [9,10,11], large oncosomes (1–10 μm) [12,13], matrix vesicles [14,15,16], migrasomes (50 nm to 3 μm) [17,18], exopheres (~4 μm), exomeres (~35 nm), and bacterial outer membrane vesicles (OMV) [19,20] [4,21]. EVs are also classified by their size into small EVs (s-EVs; 30–500 nm) and large EVs (L-EVs; >1 μm).

We have found two types of nomenclature that describe EVs and we enumerate below the terms that we use in the review, to clarify the language for the reader.

1- Although the term “exosome” has been frequently used to describe all vesicles released by cells into the extracellular milieu, it is now known that there are multiple different types of EVs, of which exosomes are only one sub-type. Distinguishing between different vesicle-subtypes within a population-mixture is very difficult, as they have overlapping compositions, densities, and sizes in addition to the lack of specific markers to differentiate the subtypes. Therefore, the International Society for Extracellular Vesicles (ISEV) proposed the use of the term EVs be used preferentially to describe vesicles prepared from body fluids and cell cultures [4].
2- EVs are composed of heterogeneous populations, and there is no unanimous consensus on the nomenclature to be used for them. General terms such as “exosomes” and “microvesicles” have been broadly used. Here we will retain the use of the traditional nomenclatures of the EVs, including exosomes, ectosomes, and oncosomes, depending on the context of the study.

Exosomes are vesicles of endosomal origin. They are initially formed as internal luminal vesicles (ILVs) in multi-vesicular bodies (MVBs) by the endosomal sorting complex required for transport (ESCRT) machinery, in ESCRT-dependent or ESCRT-independent mechanisms [22,23,24,25,26]. Firstly, the proteins are internalized from the cell surface (as with activated growth factor receptors) or transported from the Golgi network (for instance MHC class-II molecules). In order to be targeted into the vesicles, many proteins are ubiquitylated at their cytosolic domains, although not all proteins required such ubiquitinylation [27,28,29]. After vesicle accumulation, the MVBs either fuse with lysosomes to be degraded or are released as exosomes into the extracellular space [22,23,24,25,26]. These vesicles can play roles in: (1) discarding unfavorable molecules from cells and also in (2) cell-to-cell communication by transferring their cargo molecules to recipient cells or organs in local and/or distant tissues [30]. Recent studies have shown that anti-cancer drugs, including chemotherapeutics and targeted drugs, can be released from cells within EVs, suggesting a novel mechanism of drug resistance. EV-mediated drug efflux is often coupled with cellular dedifferentiation involving activation of epithelial-to-mesenchymal transition (EMT) [31]. EMT involves a cellular transformation or dedifferentiation from an epithelial phenotype into a mesenchymal phenotype and is important in many aspects of cell biology, including tissue development, inflammation, and cancer progression [32,33,34]. Epithelial cells are usually tightly connected to each other through intercellular adhesion and cell junctions including the adherence junction, desmosomes, gap junctions, synaptic junctions, and occluding/tight junction, whereas loss of these connections/adhesions in EMT is accompanied by altered cellular shape, increased motility, and migratory activities of the cells. Pre-cancerous cells often exhibit EMT, increased migration, and invasion of the cells within the tumor milieu [35]. EMT is a complex process consisting of multiple sequential steps and pathways, triggered by extracellular prompts such as transforming growth factor β (TGFβ) signaling [36], epidermal growth factor (EGF) signaling [31,37], matrix metalloproteinases (MMPs) [38], intracellular signals, and transcription factors [35]. It has been shown that EMT increases the properties of cancer stem cells (CSC) or cancer-initiating cells (CIC), which are highly resistant to therapy, recurrent after treatment, and metastatic [39,40,41].

**Figure 1:**
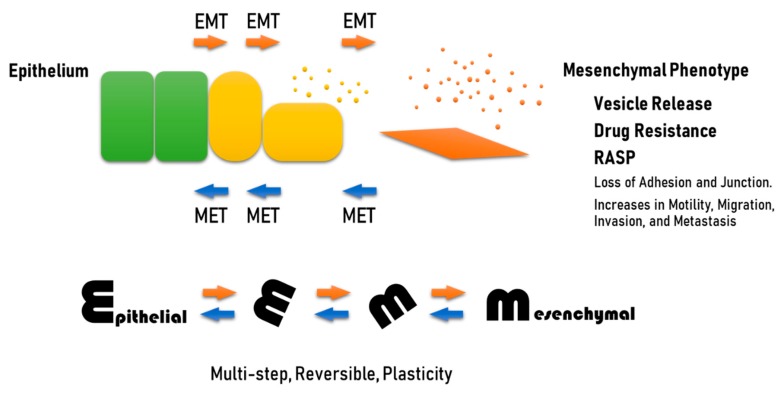
EMT, a process coupled with vesicle release, drug resistance, and RASP. EMT is a reversible process, which means that although epithelial cells can be switched into a mesenchymal phenotype (EMT), such cells can also revert to epithelial cells by undergoing mesenchymal-epithelial transition (MET). Both EMT and MET occur during normal development and during cancer progression. Epithelial cells adhere to each other through epithelial intercellular adhesion molecules (i.e., E-cadherin and claudins) and desmosomes, which are often lost in EMT. During EMT, cells become motile and acquire invasive capacities.

Recent studies have shown that increased EV release can be coupled with EMT (Figure 1). EMT enhances the EV-releasing phenotype of cells, while, conversely, tumor-derived EVs such as oncosomes initiate EMT in epithelial cells as well as driving EMT in cancer cells [31]. Among various classifications of EVs, oncosomes have been shown to promote steps in tumor progression such as EMT by transferring oncogenic molecules [31,42,43,44,45,46,47]. Moreover, anti-cancer drugs can be released with exosomes from tumor cells, suggesting a mechanism of cancer drug resistance. The vesicle-releasing and drug-releasing phenotypes can be an aspect of the resistant-associated secretory phenotype (RASP). Studies showing EMT-coupled exosome release are reviewed here as discrete mechanisms of drug resistance and immunosuppression in cancer. This review concludes that EMT is often coupled with vesicle release, drug resistance, and RASP.

## 2. EV-Mediated Oncogenesis

### 2.1. Oncosomes

Oncosomes have been defined as oncogenic EVs or oncogenic exosomes that mediate transfer of tumor-promoting factors such as oncoproteins, oncomiR, and circulating tumor DNA (ctDNA) [11,12,48,49]. The term “oncosome” was first defined by Rak et al., who reported transfer of the oncogenic receptor, EGF receptor variant III (EGFRvIII), by microvesicles secreted from brain tumor cells [11]. Next, Di Vizio et al. reported oncosome formation in prostate cancer to be associated with a region of frequent chromosomal deletion in metastatic disease [49]. This group then reported that oncosomes larger than 1 μm could be selectively sorted by flow cytometry in human prostate cancer tissues and in the circulation of mice with metastatic disease and contained MMPs, RNA, caveolin-1, and the GTPase ADP-ribosylation factor 6 (ARF6) [13]. The large oncosomes (a type of L-EVs) carried most of the tumor DNA in the circulation of prostate cancer patients [12]. In this study, whole-genome sequencing revealed that the DNA in L-EVs reflects the genetic aberrations of the cell of origin, including copy number variations (CNV) of genes frequently altered in metastatic prostate cancer, such as MYC, AKT1, focal adhesion kinase [FAK, also known as protein tyrosine kinase 2 (PTK2)], KLF10, and PTEN. Later studies have shown that a number of additional oncogenic factors were contained in oncosomes, such as oncomiR miR-520g [50], 14-3-3 and β-catenin [51].

A further proteomic study has revealed that oral cancer-derived oncosomes contain heat shock protein (HSP) family members, a number of extracellular matrix molecules (ECM), and transcriptional regulators [52]. HSPs have been shown to assist in the folding of oncoproteins essential for cancer cell survival and resistance [53,54,55]. Therefore, HSP-rich oncosomes and their molecular transfer can be crucial in tumor progression and resistance, as discussed later in more detail (Section 3.1).

### 2.2. Stroma-Derived EVs in Tumor Progression

Growing evidence indicates that tumor tissues constitute more than an accumulating mass of homogeneous cancer cells. Indeed, malignant cells are able to effectively recruit stromal cells [56], including vascular cells [57] and immune cells [58]. The recruitment of these cells involves the secretion of stimulatory growth factors, chemokines, and cytokines at the primary tumor site. Infiltrating normal cells are involved in constructing the tumor structure and build the tumor microenvironment that actively contributes to cancer progression by promoting angiogenesis, metastasis, and suppression of the anti-cancer immune response [59,60]. These cells in the tumor microenvironment include cancer-associated fibroblasts (CAFs) with properties differentiated from mesenchymal stem cells (MSCs) [61], tumor-associated immune cells including CD11b+ immune cells such as tumor-associated macrophages (TAMs) [62,63,64] and myeloid-derived suppressor cells (MDSCs) [65], tumor-infiltrating dendritic cells (DCs) [66], monocytes [67], T cells including cytotoxic T lymphocytes (CTLs) and tumor-infiltrating regulatory T cells (TITreg) [68], B cells [69], tumor endothelial cells (TECs) [70,71], adipocytes [72,73,74,75], and normal epithelial cells [76]. Such stromal cells communicate with each other and tumor cells using cytokines, growth factors, MMPs, ECM, microRNAs, and EVs. Earlier studies suggested that the tumor stroma could be tumor-suppressing, whereas recent studies show that stromal signals often drive tumor progression.

Among the various stromal cells, we here review the crucial roles of CAFs, TAMs, and TECs. It has been reported that CAF-derived cytokines and growth factors, including TGFβ, hepatocyte growth factor (HGF), FGF, NGF, IGF, and interleukin-6 (IL-6), promote cell proliferation and migration [56,77]. CAFs also enhance cell motility and EMT by stimulating cyclooxygenase 2 (COX-2)/prostaglandin E2 (PGE2) cascade and by producing TGFβ [61,78]. CAFs enhance angiogenesis by producing growth factors such as VEGF, PDGF, HGF and chemokines, such as CXCL8 (also known as IL-8) and CXCL12 [also known as stem cell-derived factor 1 (SDF-1)], which act on TECs [70]. CAF-derived CXCL12 binds its receptor CXCR4 on TECs and thus promotes angiogenesis [71]. CAFs also provoke inflammation by producing IL-6, IL-1, and adenosine triphosphate (ATP), while such cells alter macrophage polarity and elicit immune evasion by stimulating the COX-2/PGE2 cascade and by producing IL-6 and SDF-1/CXCL12. CAFs also control ECM deposition and remodeling by producing fibronectin, type-I collagen, tenascin C, osteopontin, and MMPs [79]. CAF-derived EVs carry TGFβ, MMPs, microRNA, and ECM molecules which alter the properties of epithelial cells, tumor cells, and the tumor milieu [79,80,81,82,83]. Proteomic analysis of stroma-derived EVs is important to elucidate the mechanism of the elicited tumor progression. Proteomic profiling of secretory factors (EVs and non-EV soluble factors) derived from CAFs identified 4247 proteins, among which a new cancer biomarker MFAP5 was discovered [84]. TAMs produce multiple immunomodulatory lipids, and several proteins involved in lipid metabolism were enriched in TAM-EVs, compared to source TAMs [85]. Cianciaruso et al. inoculated colon adenocarcinoma (MC38) and murine breast cancer cells (E0771) subcutaneously in C57BL/6 mice, and they found that TAM-EVs isolated from tumor-bearing mice contained bioactive lipids and biosynthetic enzymes of the arachidonic acid pathway such as COX1, thromboxane-A synthase (TBXAS1), and some CYP proteins, which redirect the catabolism from a COX2-dependent pathway toward a COX1-dependent pathway to limit the pro-tumoral effects of some prostaglandins. Thus, although TAMs exhibit pro-tumor effects, their EVs might induce tumor immunosuppression [85].

Stromal cells, including TAMs and CAFs, are also involved in drug resistance. CAFs are intrinsically resistant to cisplatin and participate actively in promoting head and neck cancer (HNC) cell survival and proliferation by transferring functional miR-196a to tumor cells via exosomes. Exosomal miR-196a binds to novel targets such as cyclin-dependent kinase (CDK)N1B and ING5 mRNA to endow HNC cells with cisplatin resistance property [80].

## 3. Resistance-Associated Secretory Phenotype (RASP)

### 3.1. HSP as Mediators of RASP

Tumor cell populations are often exposed to stresses such as immune/inflammatory stress, therapeutics [86], hypoxia, acidification and oxidative stress [87,88,89], starvation [90], nutrient stress [91], heat and cold [53,92], thermal stress, replication stress [93], ER stress, neurotoxic stress [94], genotoxic (DNA damage) [95] and proteotoxic stress [96,97]. Heat shock proteins (HSPs), originally found to be induced upon heat shock, protect from many of these stresses [53,55,98,99,100]. Subsequent studies have revealed that other types of stresses can also induce HSPs, including hypoxic stress [101] and nutrient starvation [90]. HSPs are intracellular molecular chaperones that assist in protein folding and re-folding in cells, play stress-resistance roles as anti-apoptosis factors [53], and modulate the effects of radiation therapy, chemotherapy, and immunotherapy. HSPs are increased in many types of tumor cells and play roles in tumor progression, supporting migration, invasion, and metastasis [54,55,99,102,103,104].

**Figure 2:**
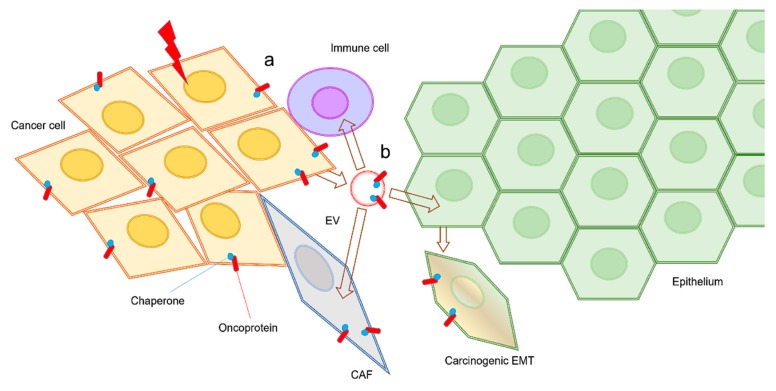
Exosome-mediated carcinogenic transfer. (**a**) Cancer cells (orange diamonds) and/or CSCs also known as CICs can express oncoproteins (red bars) such as mutant or amplified receptor tyrosine kinases (RTKs) including EGFR family members, which are functionalized by molecular chaperones HSPs (blue balls). (**b**) The carcinogenic and resistance factors of EVs can be transferred to epithelial cells (green hexagons) and initiate carcinogenic EMT [31]. These factors carried by EVs can be transferred to and alter CAFs (gray diamond) and immune cells (shown in purple) such as TAMs. The EV-mediated transfer of carcinogenic factors and HSPs is a novel mechanism of cancer expansion and malignant conversion of the tumor microenvironments leading to a resistant phenotype.

In addition, extracellular HSPs have been identified, evidently after release from cells, either within vesicles or by pathways of non-vesicular HSP secretion [52,102,105,106]. Notably, it has been shown that HSPs and vesicles were co-released upon cell stress and cell damage such as molecular targeted therapeutic stress [37,107], anti-cancer therapeutic DNA damage stress [108,109,110], and heat shock [111,112]. Extracellular HSPs and HSP-rich EVs can promote cancer progression by enhancing EMT, migration, invasion, heterogeneity, metastasis, CSC/CIC properties, and drug resistance in cancer cells and angiogenesis [113,114,115,116,117,118,119]. Proteomic analysis of oral cancer-derived oncosomes revealed a number of HSP family members to be contained within EVs, including HSP90 homologs, large HSPs, and HSP70 family members [52]. The HSPs and oncoproteins contained within EVs could be involved in RASP, co-transferred to recipient cells leading to cancer expansion, and malignant conversion of the tumor microenvironment (Figure 2) [102,120,121,122]. HSPs are often carried as cargo by EVs, including exosomes, ectosomes, and oncosomes and have also been shown to be associated on the membrane surfaces of EVs [52,102,105].

Since HSPs promote stress-resistance, secreted HSPs are a major aspect of RASP. We hypothesize that exosomal HSPs may promote the folding of oncoproteins upon molecular co-transfer to recipient cells and resultant increases in chaperoning power. Several aspects of RASP including HSP mediators and oncosomal molecular cotransfer of oncoproteins are extensively discussed in our recently-published review [102]. Indeed, highly metastatic oral cancer-derived s-EVs contained significant levels of HSPs, including HSP90α, HSP90β, TRAP1, HSP110/HSPH1, and HSP70, which were coordinately increased with EGFR and CD326 (also known as an epithelial cell adhesion molecule (EpCAM)) as compared with low metastatic cell lines [52]. Oncosomal molecular cotransfer of oncoproteins such as mutant EGFR and amplified HSPs [123] can thus promote oncogenesis and resistance to stress and therapy in cancer cells themselves and in the recipient cells at the local and distant milieu [31,48,52].

As mentioned above, many members of the HSP family play key roles in cell survival and the promotion of drug resistance [53,102,124,125,126,127]. Extracellular HSPs and EVs enriched with such cytoprotective HSPs are thus a major aspect of the RASP. Molecular transfer of HSPs may increase drug resistance in cancer cells and influence the tumor microenvironment. Heat shock factor 1 (HSF1) is a master transcription factor for the stress response and induction of HSPs [55,96,100,128,129,130]. The HSF1-HSP transcriptional system is a key axis in the stress response as well as in the stress resistance of cancer cells.

### 3.2. Exosomal Ejection of Drugs

**Figure 3:**
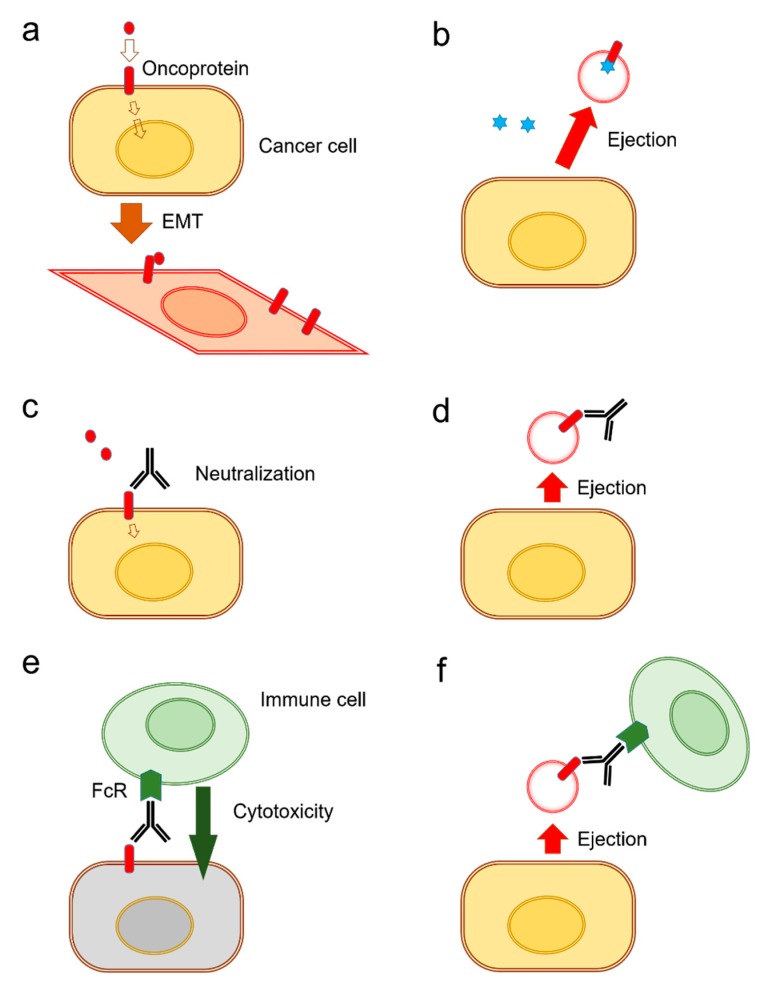
Exosome release mechanisms for the ejection of drugs and antibodies. (**a**) Cancer cells and CSCs/CICs express oncoproteins, such as mutant or amplified EGFR, whose transmembrane signaling promotes the progression of cancer cells, e.g., EMT [31]. Yellow: a round-shaped cell, before EMT. Red: a diamond-shaped cell, after EMT. (**b**) Cancer cells can eject molecularly-targeted drugs (blue stars). (**c**) Neutralization of ligand-receptor interaction is a major mechanism of action of antibody-based agents. Activities of cell-surface oncoproteins can be neutralized and inhibited by host-derived endogenous antibodies and molecularly-targeted antibody drugs [37]. (**d**) Cancer cells can eject molecularly-targeted antibodies by releasing exosomes that contain oncoproteins as RASP [37]. Meanwhile, cancer cells can further transform and acquire resistant phenotypes. (**e**) The antibodies are also recognized by FcR of immune cells, including phagocytes and NK cells. (**f**) Cancer cells can also become resistant to immune cells by releasing EVs.

It has been shown that the release of exosomes is often coupled with EMT in tumor cells [31] (Figure 3a). There are currently two known types of EV-mediated (or exosomal) mechanisms of anti-cancer drug ejection. In the first mechanism, chemotherapeutics are secreted when enclosed within exosomes (Figure 3b). Indeed, it has been reported that cisplatin was secreted in exosomes from ovarian cancer cells [131], melanoma cells [132], and A549 lung cancer cells [133] (Table 1).

The second mechanism is EV-mediated ejection of drugs that target cell surface molecules such as EGFR-targeted cetuximab resistance [37]. Antibody-based therapeutics are able to neutralize receptor-ligand interactions (Figure 3c). However, such antibody-based medications can be released along with exosomes from the cells (Figure 3d). Indeed, the targeted anti-EGFR antibody medication cetuximab binds to EGFR on the cell surface and inhibits receptor-mediated EMT [31]. However, cetuximab was ejected by oral cancer cells in EVs that contained EGFR in response to therapy [37]. Cell surface oncoproteins, such as CD326/EpCAM, EGFR, and programmed cell death-ligand 1 (PD-L1), are often released from cancer cells by two mechanisms including secretion in exosomes and protein shedding by proteinases. The oncosomes containing such cell surface molecules can play roles as decoys against molecularly targeted drugs. Secondly, in antibody-dependent cellular cytotoxicity (ADCC), antibody drugs can recruit Fragment crystallizable region receptor (FcR)-expressed immune cells leading to cytolysis by CTLs or by natural killer (NK) cells and phagocytosis by macrophages (Figure 3e). However, these antitumor immune cells can be released with EVs from cancer cells (Figure 3f). EV-mediated ejection of drugs is a novel mechanism of drug resistance in cancer cells as well as a novel aspect of RASP.

### 3.3. Extracellular Vesicles as Immunosuppressive Agents

A variety of immune cells infiltrate the tumor microenvironment as part of immune surveillance. However, tumor cells secrete immunosuppressive cytokines that counteract effective immune responses in order to promote cancer cell survival and proliferation. Many tumor-derived EVs are rich in the pro-apoptotic Fas ligand (Fas-L). Fas-L-enriched tumor-derived EVs induce the apoptosis of anti-tumor effector CD8+ T cells, as well as promoting the expansion of T regulatory cells, consequently contributing to immune suppression [139]. Notably, the induction of T-cell apoptosis by Fas-L-containing EVs has been reported in several cancer models, including melanoma, prostate cancer [140], colorectal cancer [141], and HNC. Other mediators such as galectin-1 and -9 in tumor-derived EVs were also found to induce T-cell apoptosis and immune suppression within the tumor [142,143]. Moreover, tumor-derived EVs enriched in TGFβ have been shown to inhibit IL-2-induced T-cell proliferation and to induce regulatory T cell (Treg) phenotype in acute myeloid leukemia (AML) [144], mesothelioma [145], and colorectal cancer [146]. Tumor-EVs also impaired monocyte differentiation into dendritic cells and promoted MDSC generation [147]. It has additionally been shown that treating NK cells with exosomes containing MHC class 1 related chain ligand A (MICA) triggered the downregulation of the NK receptor NKG2D and provoked a marked reduction in NK cytotoxicity independent of NKG2D ligand expression by the target cell [148].

DCs are the most potent antigen-presenting cells that prime antitumor immunity [149]. However, tumor cells can domesticate these cells, and alter their maturation and activation through galectin-1 and IL-6 to confer a pro-tumorigenic phenotype that promotes tumor growth, metastasis, and immune escape [150]. Shen et al. have demonstrated that HSP72 and HSP105-enriched exosomes could educate dendritic cells to promote tumorigenesis and led to the induction of IL-6 secretion in a toll-like receptor (TLR)2- and TLR4-dependent manner. These effects dramatically promoted tumor invasion by increasing MMP-9 metalloproteinase transcription in tumor cells [151].

**Figure 4:**
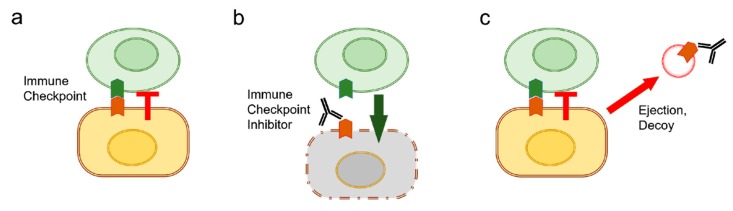
Role of EV release in immune evasion. (**a**) The immune checkpoint enables cancer cells to evade killing by cytotoxic T cells. For example, cancer cells express PD-L1 (shown in dark brown) that binds with PD-1 (shown in dark green) on the surface of immune cells and acts as a co-inhibitor of the T cell receptor. (**b**) Immune checkpoint inhibitors can block the checkpoint and enable immune cells to attack cancer cells. (**c**) However, cancer cells could eject immune checkpoint inhibitors by releasing EVs that contain checkpoint proteins.

PD-L1 is an immunosuppressive molecule that is mainly expressed on the surfaces of tumor cells [17]. The interaction of PD-L1, with its receptor programmed death 1 (PD-1), inhibits T cell-mediated cellular immune responses, including priming, growth, proliferation, and apoptosis, and functional maturation [18] (Figure 4a). Recent studies have shown that the activation of the PD-1/PD-L1 signaling pathway inhibits T cell responses through inducible Tregs (iTregs) [152], as well as mediating the arrest of the T cell cycle at the G1 phase [153]. Disrupting the interaction of the PD-L1 ligand with the PD-1 receptor on T cells restores T cell-mediated immune responses and potentiates anti-tumor immunity (Figure 4b). However, not all patients respond to such immune checkpoint inhibitors, as exosomes secreted by tumor cells carry bioactive PD-L1 on their surface and can thus suppress the immune response [76] (Figure 4c). EVs were found to capture the anti-PD-L1 antibody and display it on their surface thereby engaging with PD-1 on tumor-specific T cells [154]. Likewise, glioblastoma tumor-derived EVs were shown to express PD-L1 and to inhibit T cell proliferation and antigen-specific T cell responses in vitro [155]. We show a model depicting how immune checkpoint inhibitors could be ejected by cancer cells through the release of the exosome/PD-L1 antibody complex in Figure 4.

### 3.4. Release of Oncogenic Lipids and Lipophilic Drugs

The balance of metabolite homeostasis is often disrupted in cancer cells, a phenomenon driven by oncogenic signaling or genetic mutation of critical metabolic enzymes [156]. The accumulation of certain lipid species due to the aberrant activity of lipid metabolism may also be a causal factor in tumor malignant progression and metastatic behavior [157]. Increases in signaling lipids, including eicosanoids [such as prostanoids, leukotrienes (LTs), epoxyeicosatrienoic acids (EETs)], phosphoinositides, sphingolipids, and fatty acids (FAs), alter the behavior of cells and might be a causal factor in tumor malignant progression and metastasis. For example, a radiation-induced phenotype in mammary carcinoma cells involving the acquisition of enhanced migratory and metastatic properties required increased activity of COX2 and the activity of PGE2 receptor EP4 [95]. Such a malignant phenotype was cumulative with damage, and levels of stem cell markers, including stem cell antigen-1 (Sca-1; also called Ly6a) and aldehyde dehydrogenase (ALDH1), increased with treatment dose. The Sca-1+ metastatic phenotype was inhibited by both COX2 inhibitors and PGE2 receptor antagonists [95]. In addition to the COX2/prostaglandins cascade, other eicosanoids such as LTs and EETs, FAs, fatty acid-binding proteins (FABPs), and sphingolipids, are signaling lipids that induce malignant phenotypes, including EMT, CSC/CIC pool, circulating tumor cells (CTCs), and metastatic dissemination of exosome/ oncosome [157,158,159]. Moreover, the transfer of acid sphingomyelinase (ASM) contributes to drug resistance in multiple myeloma (MM) [160]. ASM was increased in response to anticancer drugs (melphalan or bortezomib) in MM cells and their exosomes. ASM-high exosomes were able to transfer the drug-resistant phenotype to chemosensitive cells, thus suggesting a tumor-protective role for ASM. The drug-resistant phenotype of MM cells was inhibited by amitriptyline, an inhibitor of ASM and monoamine transporters for serotonin, norepinephrine, and dopamine [160,161,162]. Thus, EV formation and secretion mediated by lipids may be essential for the promotion of tumor invasion and metastasis.

Lipid efflux is also an aspect of RASP. EVs are surrounded by lipid bilayers. Redundant lipids can be evicted from cells through the release of lipid-layered EVs and lipid cholesterol efflux pumps, such as ATP-binding cassette (ABC) transporters. One such lipid efflux pump that is overexpressed in metastatic cancer cells is ABC-G1 [163]. siRNA-mediated silencing of ABC-G1 triggered the accumulation of EV lipid and cell death in tumoroids, suggesting that tumor cells may release unfavorable lipids as a cell survival strategy. Most of the ABC members transport lipophilic substrates such as phospholipids and include ABC-A1, A3, A4, A7, A12, B1, B4, and C1; sphingomyelin transported by ABC-A1 and A3; sphingolipids by ABC-B1; cholesterol by ABC-A1, A2, A5, G1, G4, and G5/G8; bile salts by ABC-B11; drugs transported by ABC-B1, C1, C2, and G2, steroids transported by ABC-C1, C10, G2, and G5/G8; and very-long-chain fatty acids (VLC-FAs) by the ABC-D group (D1 to D4) [164]. Notably, most drugs have been designed to possess lipophilic properties in order to cross the lipid-rich cell membrane and enter the cytoplasm. However, resistant cancer cells may eject such lipophilic drugs using ABC family transporters and lipid vesicles.

## 4. Exosomal Drug Resistance

Cancer cell drug resistance is one of the greatest hurdles in tumor treatment. Recently, EVs have emerged as important modulators of drug resistance through different mechanisms that impair drug efficacy [165,166]. Several studies reported that platinum drugs are released with exosomes from cancer cells. The antibody-drug cetuximab was also ejected with s-EVs by cancer cells [37]. Exosome-release is often coupled with EMT phenotypes in cancer cells. The resistance of malignant cells to different classes of anticancer drugs is termed multidrug resistance (MDR), a property that can be acquired by several mechanisms including: (1) altered cellular proliferation, (2) increased DNA repair capability, (3) decreased susceptibility to apoptosis, (4) alteration of drug targets, (5) overexpression of MDR proteins, and (6) increased drug export [165,166]. EVs play a crucial role in mediating intercellular communication by transferring nucleic acids and proteins from the donor cells to remote recipient cells; tumor cells can shed greater amounts of EVs than normal cells, and these EVs are most likely relevant for the transfer of the drug-resistant trait [167]. Indeed, tumor-MDR cells release more microvesicle-like EVs and fewer exosomes compared to their drug-sensitive counterpart cells that produce more exosomes [168]. More interestingly, the EVs derived from the MDR cells can induce chemoresistance in chemosensitive cells [169,170,171,172]. Consequently, these drug resistance cargos, delivered by the EVs, contributed significantly to the development of drug resistance.

EVs can mediate drug resistance by one of at least three different mechanisms: (i) by sequestering cytotoxic drugs, thereby decreasing the effective drug dose within the target sites below the concentration required to produce the desired therapeutic effect [166], (ii) via the direct transfer of MDR proteins from resistant tumor cells to sensitive cells [173]. Cancer cells export drugs into the extracellular milieu using the MDR-ABC transporters system, thus preventing the intracellular accumulation of many anti-cancer drugs and diminishing drug efficacy [174]. Shedden et al. were the first to demonstrate the direct correlation between the drug resistance and expression of genes associated with vesicle shedding in different cancer cell lines [175]. Their study revealed that breast cancer cells encapsulated doxorubicin into vesicles and expelled the drug into the extracellular milieu [175]. Furthermore, melanoma [132] and ovarian carcinoma cells [131] resisted cisplatin therapy by directing its export into these vesicles and increasing the EVs’ secretion. Moreover, EVs carrying the P-glycoprotein (P-gp, also called MDR-1 or ABCB1) drug efflux pump mediated the transfer of multidrug resistance to sensitive cells in many human cancer models, including prostate and ovarian cancers, acute T lymphoblastic leukemia, and osteosarcoma [169,171,172,173]. In mechanism (iii), EV-mediated export of bioactive cargoes (such as prosurvival factors, apoptosis inhibitors molecules, and non-coding RNAs) reprograms the cell cycle and apoptosis in recipient cells [166]. For instance, TGF-β1 was enriched in tumor-derived EVs and was reported to induce regulatory T cells and to inhibit the proliferation of peripheral blood lymphocytes from healthy donors in response to IL-2 [27]. Additionally, in vivo and in vitro resistance to sorafenib in hepatocellular carcinoma cell lines was induced by the EV-mediated delivery of HGF and subsequent HGF/c-MET/PI3K/AKT signaling pathway activation, a major oncogenic signaling axis involved in cancer cell proliferation and survival [176]. Furthermore, survivin, a member of the Inhibitors of Apoptosis (IAP) family, was enriched in EVs secreted from different tumor types [177,178,179]. This protein has been implicated in suppressing cell death and promoting mitosis [180]. Indeed, treating highly aggressive MDA-MB-231 breast cancer cells with Paclitaxel promoted the secretion of EVs enriched with survivin that significantly contributed to the survival of serum-starved and Paclitaxel-treated fibroblasts and SKBR3 breast cancer cells [181]. Additionally, a recent study has demonstrated that the level of plasma gelsolin (pGSN), an actin-binding protein [182], is abundantly expressed and secreted in chemoresistant ovarian cancer cells compared to its chemosensitive counterparts. Furthermore, gain- and loss-of-function studies showed that pGSN confers cisplatin resistance to ovarian cancer cells. Exosomal pGSN was upregulated via the α5β1 integrin-FAK-Akt-HIF1α signaling pathway and thus inhibited cisplatin-induced apoptosis. Exosomal pGSN derived from chemoresistant cells provoked cisplatin resistance in chemosensitive target cells through exosomal uptake and accumulation of pGSN. The high expression level of pGSN in ovarian cancer patients significantly correlates with poorer overall survival and relapse-free survival [183].

Recently, it has been found that the transfer of miR-365 in EVs derived from macrophage prompts the resistance of pancreatic adenocarcinoma cells to gemcitabine in vitro and in vivo [63]. Similarly, miR-21 transferred from cancer-associated adipocytes and fibroblasts to ovarian cancer cells to reduced apoptosis and induced resistance to paclitaxel by downregulating the mRNA expression of apoptotic peptidase activating factor (APAF1) [184]. miR-21-derived TAMs were shown to activate PI3K/A, suppress apoptosis, and induce cisplatin resistance in gastric cancer cells [185]. Detailed mechanisms of EV-mediated drug resistance are reviewed in Maacha et al. [166].

Several studies have reported that platinum drugs such as cisplatin and carboplatin were released with exosomes, mediating chemoresistance in cancer cells (Table 1). In addition, cetuximab, an anti-EGFR antibody medication, was also released with oncosomes [37]. Cancer cells found in HNC, colorectal carcinoma, and non-small cell lung carcinoma (NSCLC) often acquire genetic amplification of EGFR, and EGFR-containing EVs are released from these cancer cells. Cetuximab bound to EGFR-EVs was co-released from HNC cells, suggesting a mechanism of cancer drug resistance [37]. Interestingly, recent studies have shown that vesicle-releasing properties are often coupled with cellular transformation phenotypes, including EMT [31,37,135,138] and CSC [106,136]. Thus, it is conceivable that the mesenchymal transition and CSC phenotype are involved in the acquisition of exosome/drug-releasing properties. This concept is supported by the oncogenic role of Src in both exosome secretion and EMT. It was recently shown that the oncoprotein Src in endosomal membranes promoted exosome secretion and tumor progression [186]. Consistently, Src promotes EMT triggered by multiple EMT inducers including EGF [187], leptin [188], Cten [189], and δNp63γ [190]. Anti-EMT strategies involving targeting the TGFβ receptor or CDK2 may inhibit exosome/oncosome release from cancer cells [36].

EMT, almost by definition, enhances the motility and migration of tumor cells. It has been recently shown that cells release migrasomes during cell migration from cellular cilia, the tail of the cell [17,191]. The proteome of migrasomes was 27% in common with exosomes, while the remaining 73% was specific to migrasomes [191]. These findings prompted us to hypothesize that EMT-driven migration of cancer cells may promote the release of EVs, which enhances drug ejection and resistance in malignant cells.

On the other hand, it has been shown that drug-encapsulated exosomes derived from immune cells and MSCs can be effectively and efficiently delivered to cancer cells. Indeed, macrophage-derived exosome-encapsulated paclitaxel was developed to overcome MDR in cancer cells [192]. Targeted delivery of a TLR3 agonist with single-chain antibody fragment-conjugated nanoparticles induced a type I-interferon response and apoptosis in tumor cells [193]. As these are interesting but complex aspects of the topic, we will discuss them more extensively in the future.

## 5. Conclusions

EV-mediated ejection of anti-cancer therapeutics is a novel mechanism of drug resistance that can develop in cancer. Chemotherapeutics, as well as antibody drugs, can be released with EVs derived from the tumor cells. EV/drug-releasing phenotypes are often coupled with cellular transforming processes such as EMT and CSC/CIC. RASP is a marker of resistant phenotypes and a potential target to inhibit EV release from cancer cells.

## Figures and Tables

**Table 1:** Exosomal drug resistance.

Microenvironment	Determinant Pathway	Phenotype	Type of Resistance	Reference
**Hypoxia**	STAT3 Rab27↑ Rab7↓ Lamp1/2↓	Exosome release Secretory lysosome	Platinum resistance	[134]
**Hypoxic tumoroids**	EpCAM-exosome Extracellular HSP90α	CSC Exosome release	CSC/CIC phenotype	[106]
**Extracellular Acidosis (Low pH)**	Proton pump	Exosome release	Platinum resistance	[132]
**TGFβ signal**	Smad4 mutation	EMT Exosome release	Platinum resistance	[135]
**EGF signal**	EGFR amplification EGFR-exosome	EMT Exosome release	Cetuximab resistance	[31,37]
**Stromal fibroblasts**	Wnt-exosomes	CSC Exosome release	Chemoresistance	[136]
**-**	HSP90-exosome	Exosome release	Anti-apoptotic Survival of metastatic cancer cell	[52,105]
**-**	HSF1/HSPs	EMT ECM remodeling	Radioresistance Chemoresistance	[137]
**-**	miR-155-5p GATA3↓ TP53INP1↓	EMT Exosome release	Paclitaxel resistance	[138]

## Abbreviations

ABC	ATP-binding cassette
ADCC	Antibody-dependent cellular cytotoxicity
Akt	A serine/threonine-specific protein kinase
ATP	Adenosine triphosphate
CAF	Cancer-associated fibroblast
CDK	Cyclin-dependent kinase
CIC	Cancer-initiating cell
COX	Cyclooxygenase
CRPC	Castration-resistant prostate cancer
CSC	Cancer stem cell
CTL	Cytotoxic T-lymphocyte
CYP450	Cytochrome P450
ECM	Extracellular Matrix
EGF	Epidermal growth factor
EMT	Epithelial to mesenchymal transition
EV	Extracellular vesicle
FcR	Fragment-crystallizable receptor
HGF	Hepatocyte growth factor
HNC	Head and neck cancer
HSF	Heat shock factor
HSP	Heat shock protein
MDR	Multidrug resistance
MMP	Matrix metalloproteinase
MSC	Mesenchymal stem cell
MZF1	Myeloid zinc finger 1
NK	Natural killer
NKG2D	Natural killer group 2 member D
OMV	Outer membrane vesicles
OSCC	Oral squamous cell carcinoma
PD-1	Programmed cell death-1
PD-L1	Programmed cell death-ligand 1
PI3K	Phosphatidylinositide 3-kinase
RASP	Resistance-associated secretory phenotype
RTK	Receptor tyrosine kinase
TGFβ	Transforming growth factor β
Tumoroid	Tumor organoid
